# Prognostic Value of Clinicopathological Parameters Assessed During Admission of Foals with Neonatal Encephalopathy and Comorbidities Developed During Hospitalization

**DOI:** 10.3390/vetsci11110534

**Published:** 2024-11-01

**Authors:** Alexandra Vilela e Maia, José Pimenta, Mário Cotovio

**Affiliations:** 1 Veterinary Teaching Hospital, University of Trás-os-Montes e Alto Douro, 5000-801 Vila Real, Portugal; alexandra.vilela@hotmail.com; 2 CIVG Vasco da Gama Research Center/EUVG, Vasco da Gama University School, 3020-210 Coimbra, Portugal; 3 CECAV—Veterinary and Animal Research Center, University of Trás-os-Montes e Alto Douro, 5000-801 Vila Real, Portugal; mcotovio@utad.pt; 4 Associate Laboratory for Animal and Veterinary Sciences (AL4AnimalS), 5000-801 Vila Real, Portugal; 5 Faculty of Veterinary Medicine, Lusófona University, 4000-098 Lisbon, Portugal

**Keywords:** foal, neonatal encephalopathy, neonatal maladjustment syndrome, perinatal asphyxia syndrome, dummy foal, prognostic factors

## Abstract

Neonatal encephalopathy represents a broad neurological syndrome which encompasses newborn foals presenting a variety of non-infectious neurologic signs and/or abnormal behaviors in the immediate postpartum period. It is recognized as the most predominant neurological disorder in neonatal foals. Prognostic factors can guide clinicians in medical decision-making, aiding in the establishment of survival probabilities. The aim of this work was to scrutinize the prognostic value of clinical and laboratorial findings upon admission and posterior comorbidities developed during hospitalization, assessing their influence on the outcome of foals with NE. For this purpose, the medical records of 61 neonatal foals with a primary diagnosis of NE were retrospectively analyzed. The overall survival rate was 57.4%. Most foals presented recumbency at admission, which was associated with higher mortality rates (*p* = 0.002). Hypothermic foals at admission were 4.85 times more likely to succumb (*p* = 0.015). The presence of hypoglycemia at admission was associated with higher mortality rates (*p* = 0.002). Foals with hypercreatinemia at admission had 6.67 times greater odds of dying. The development of seizures contributed to 4.14 greater odds of dying. Foals that developed comorbidities during hospitalization had 40.1 times greater odds of dying, with pneumonia and sepsis being the most relevant comorbidities. In foals with NE, rectal temperature, hematocrit, blood glucose, and creatinine concentrations are simple, quick-to-measure parameters that may have prognostic value during admission.

## Simple Summary

Neonatal encephalopathy (NE) is the most common neurological disorder observed in neonatal foals. Identifying prognostic factors can assist veterinarians in making informed decisions and predicting survival outcomes. This study aimed to evaluate the prognostic significance of clinical and laboratory findings at the time of admission, as well as the impact of comorbidities developed during hospitalization, on the survival of foals with NE. A retrospective analysis of medical records from 61 foals diagnosed with NE was conducted. The overall survival rate was 57.4%. Recumbency at admission was significantly linked to higher mortality rates (*p* = 0.002). Foals presenting with hypothermia were 4.85 times more likely to die (*p* = 0.015). Hypoglycemia at admission was also associated with increased mortality (*p* = 0.002). Foals that developed seizures presented a 4.14 times higher risk of dying. Additionally, foals with increased creatinine concentrations had a 6.67 times higher risk of death. The development of comorbidities, such as pneumonia and sepsis, during hospitalization increased the odds of mortality by 40.1 times. Simple clinical parameters like rectal temperature, blood glucose, and creatinine concentrations at admission may have important prognostic value in foals with NE.

## 1. Introduction

Neonatal encephalopathy (NE) syndrome refers to neonates with non-infectious neurologic signs or uncharacteristic behavior in the immediate postpartum period [1]. Although the etiology is not fully understood, it is supposed to be a consequence of several antepartum and intrapartum events that lead to cerebral hypoxia and ischemia. Furthermore, based on current comparative research, some studies showed increased progestogen and neuroactive steroid concentrations in these foals so it is reasonable to assume that they may play a role in NE [2,3,4]. Another possible theory regarding the pathophysiology of this disease has emerged from human medicine, as some cases of NE do not show evidence of a hypoxic–ischemic event. This theory suggests that a fetal systemic inflammatory response to an intrauterine infection could lead to the release of pro-inflammatory cytokines, which may cause neonatal brain injury. Since placentitis in mares is relatively common and is considered a risk factor for acute neurological dysfunction in foals, this theory seems plausible but requires further corroboration [3,5,6]. NE is considered the most prevalent neurological disease of newborn foals being a life-threatening condition [7,8,9]. No consensus exists regarding nomenclature, with other terminologies being used to describe this disease, including neonatal maladjustment syndrome, neonatal multisystemic syndrome, hypoxic–ischemic encephalopathy (HIE), perinatal asphyxia syndrome (PAS), dummy foal syndrome, wanderer, and barker foal [3,10,11,12,13].

NE is recognized across different species; however, the knowledge that exists specifically about horses is somewhat scarce, being mostly extrapolated from humans and other animals [14].

Clinically, newborn foals with NE may appear normal at birth and may develop behavioral and neurological signs hours later. Behavioral abnormalities include a lack of affinity for the mare, abnormal udder seeking and suckling, tongue incoordination, and abnormal vocalization [1,15]. Neurological disturbances can include alterations in muscle tone, changes in responsiveness, seizures, autonomic disturbances, and vestibular signs. Due to behavioral and neurological disturbances at birth, affected foals may have a decreased capability to meet their nutritional needs and antibody-rich colostrum intake, which impairs immunity, and is called failure of passive transfer immunity (FPTI) [1,9,11,12]. Under such circumstances, many of these foals begin to develop organic disturbances that can culminate in non-specific laboratory findings such as acid–base and electrolyte disturbances, failure of body temperature regulation, poor glucose control (hyperglycemia or hypoglycemia), azotemia, and high levels of blood creatinine [1,9,12,16,17]. NE may lead to compromised gastrointestinal and respiratory physiologic functions which can be complicated with systemic disease characterized by varying degrees of multiorgan dysfunction. These foals can also present concomitant sepsis as a consequence of the failure of passive transfer immunity (FPTI) [1,2,3].

Considering the high propensity for sudden deterioration of the clinical state, these foals should be closely monitored [1,18,19]. The treatment is mainly supportive, directed to control neurological disturbances and concomitant metabolic dysfunctions, with some authors defending the Madigan’s squeeze procedure for some cases [1,20,21]. Although the prognosis for foals with primary diagnosis of NE without complications is generally good (70% to 80% of survival rates), this scenario can change in the presence of clinical and laboratorial disturbances presented above or comorbidities [1]. Treatment and recovery become more difficult with guarded prognosis. However, there is a great lack of prognostic studies in foals with NE aimed at helping clinicians understand which clinical and laboratory findings most negatively influence prognosis, and which should be used to advertise owners and aid decision-making. Since these foals can carry high medical costs, a prognostic outcome is valuable, reinforcing the need for such studies.

The aim of this study was to evaluate the impact that clinical and laboratory factors assessed during admission, as well as comorbidities developed during hospitalization, have on foals with NE, and thus determine which can be used as prognostic factors.

## 2. Materials and Methods

### 2.1. Data Collection

Medical records of foals diagnosed with NE between 2000 and 2022 in the Veterinary Teaching Hospital of the University of Trás-os-Montes e Alto Douro, Portugal, were retrospectively analyzed and the clinical and laboratory information presented was collected. Not all medical reports were complete. Therefore, some foals lack clinical or laboratory data.

### 2.2. Inclusion Criteria

Newborns were included in the study if they presented signs of abnormal behavior at admission such as disorientation, abnormal udder seeking, lack of suckle, recumbency, tongue incoordination, lack of affinity to the mare, abnormal vocalization, or seizures developed during hospitalization.

### 2.3. Exclusion Criteria

Newborns were excluded from the study if they did not present any abnormal behavior at admission, if they presented severe electrolyte disturbances that could cause abnormal mentation, or when clear signs of septicemia were present at admission (sepsis was defined by a positive blood culture and a septic score > 12) [10].

### 2.4. Medical Records Information

Data from foal and dam medical history included: foal sex, foal age at admission, time since the presentation of clinical signs, foal prematurity (defined as less than 320 days of gestation and the presence of signs such as silky haircoat and floppy ears), mare age, history of dystocia, placental abnormalities, and mare’s disease during pregnancy.

### 2.5. Clinicopathological Features Collected at Admission

Rectal temperature (37–39 °C), heart rate (80–120 beats per minute), respiratory rate (30–40 breaths per minute), hematocrit (35–45%), total protein (4.0–6.6 mg/dL), blood glucose concentrations (76–131 mg/dL), blood creatinine (<2.12 mg/dL), blood urea nitrogen (BUN) (<22 mg/dL), and immunoglobulin G (Ig G) concentrations (>800 mg/dL) [12,22,23].

### 2.6. Comorbidities Developed During Hospitalization

All the medical complications observed were considered; namely, diarrhea, colic, pneumonia, sepsis, and umbilical infection.

### 2.7. Statistical Analysis

For statistical purposes, foals were divided into two groups: survivors and non-survivors. The group of “non-survivors” included all the foals that died naturally due to the disease or consequences of it, during the hospitalization. The “survivors” group included all the foals that survived and returned home. Euthanized foals were not included in the study. The variables regarding clinicopathological features collected at admission and comorbidities described before were compared between survivors and non-survivors.

The Shapiro–Wilk test was performed to assess if continuous variables followed the normal distribution. The student’s t-test was performed for variables that followed the normal distribution and the Mann–Whitney test was performed for variables that did not follow the normal distribution.

Categorical variables were evaluated with chi-square and Fisher exact tests and, when applicable, an odds ratio was performed.

A binomial logistic regression model was employed to assess the impact of various parameters on the survival of foals with NE.

All the statistical analysis was considered significant when *p* < 0.05. The statistical analysis was performed using Jamovi 2.3.2.

## 3. Results

### 3.1. Sample Characterization

This study comprised 61 foals, of which 29 (47.5%) were colts and 32 (52.5%) were fillies. The overall survival rate was 57.4% (*n* = 35 survivors), with a total of non-survivors of 42.6% (*n* = 26). No association between sex and survival was found (*p* = 0.651).

### 3.2. Foals and Mares’ Medical History

Thirteen foals (21%) were premature, of which five survived and eight died. Forty-eight foals (79%) were non-premature, of which 30 survived and 18 died. However, premature birth showed no association with survival (*p* = 0.209).

According to the mares’ medical history, 17/61 of the births were dystocia; only three mares presented placental abnormalities and only five mares had a history of disease during pregnancy. None of the information regarding foals or dam medical history presented any kind of significant association with survival (Table S1).

### 3.3. Clinicopathological Features

#### 3.3.1. Rectal Temperature

A total of 18/52 (34.6%) foals were normothermic (reference range: 37 °C–39 °C), 31/52 (59.6%) were hypothermic, and 3/52 (5.8%) were hyperthermic. For statistical purposes, we excluded the hyperthermic foals (*n* = 3) from the sample which allowed us to perform an odds ratio analysis. There was a statistically significant association between rectal temperature and survival (*p* = 0.015) (Table 1). Foals with NE suffering from hypothermia at admission were found to have 4.85 greater odds for non-survival. There was also a significant difference (*p* = 0.04) in the rectal temperature means between survivors (37.2 ± 2.2 °C) and non-survivors (35.2 ± 2.07 °C).

#### 3.3.2. Heart Rate

A total of 28/47 foals had a heart rate within the reference range (reference range: 80–120 beats per minute), 8/47 had bradycardia, and 11/47 had tachycardia, upon admission. No association with survival was detected (*p* = 0.52) and there were no significant differences in the heart rate means between survivors (99.2 ± 29) and non-survivors (100 ± 24.7) (*p* = 0.52). This information is presented in Table 2.

#### 3.3.3. Respiratory Rate

A total of 12/43 foals had a respiratory rate within the reference range (reference range: 30–40 breaths per minute), 23/43 had bradypnea, and 8/43 had tachypnea. No association between respiratory rate and survival was found (*p =* 0.14). No significant differences in the respiratory rate means were found between survivors (28.9 ± 8.24) and non-survivors (38.5 ± 23.2) (*p* = 0.115). This information is presented in Table 3.

#### 3.3.4. Hematocrit

Upon admission, 8/61 (13.4%) foals had anemia and 53/61 (86.9%) did not (hematocrit between 35–45%). Anemia was also associated with non-survival (*p* = 0.04) since most of the foals that presented with anemia did not survive (Table 4). According to the odds ratio analysis, a foal admitted with NE and concomitant anemia has 4.95 greater odds of non-survival.

#### 3.3.5. Total Protein

Upon admission, 38/47 foals had total protein concentration within normal ranges (4.0–6.6 mg/dL), 3/47 had hypoproteinemia and 6/47 had hyperproteinemia. There was no association between total protein values and survival (*p* = 0.613) (Table 5), and no differences in the means between survivors (5.55 ± 1.10) and non-survivors (5.30 ± 1.19) (*p* = 0.124).

#### 3.3.6. Recumbency

Fifty-three foals had some degree of recumbency when they arrived at the hospital, which was statistically associated with non-survival (*p* = 0.009) since 43% (*n* = 26) of the foals with recumbency died (Table 6). A foal with NE that presents recumbency at admission has a 6.84 higher likelihood of dying.

#### 3.3.7. Seizures

In total, 18/61 (29.5%) foals developed seizures during hospitalization. There was a significant association between the development of seizures and non-survival (*p* = 0.022) (Table 7), with foals that presented with seizures having a 4.14 times higher risk of dying.

#### 3.3.8. Blood Glucose Concentration

A total of 16/49 (32.7%) foals had values within normal ranges (76–131 mg/dL), 25/49 (51%) had hypoglycemia, and 8/49 (16.3%) had hyperglycemia, upon admission. Blood glucose concentration was statistically associated with survival (*p* = 0.002) (Table 8). Foals with NE presenting hypoglycemia upon admission were found to have 6.67 greater odds of non-survival. Blood glucose medians presented significant differences (*p* = 0.02) between survivors (121 (67.3) mg/dL) and non-survivors (38 (98.5) mg/dL).

#### 3.3.9. Blood Creatinine

Upon admission, 13/34 (38.2%) foals had blood creatinine concentration within the reference range (<2.12 mg/dL), and 21/34 (61.8%) had hypercreatinemia. Creatinine concentration was associated with survival (*p* = 0.013) (Table 9). A foal with NE presenting elevated creatinine concentration upon admission has 6.67 greater odds for non-survival. Survivors and non-survivors presented significant differences (*p* = 0.018) in median creatinine concentration: 1.95 (1.06) mg/dL and 3 (1.74) mg/dL, respectively.

#### 3.3.10. BUN

A total of 15/40 foals had BUN concentration within reference (<22 mg/dL) and 25/40 had elevated concentrations (Table 10). No association with survival was detected, and there was no difference in the means between survivors (27.1 ± 11.3) and non-survivors (27.6 ± 13.7) (*p* = 0.860).

#### 3.3.11. FTPI

Although 41/61 (67.2%) of foals presented FTPI upon admission, no association with survival was found (*p* = 0.793) (Table 11). All 41 foals diagnosed with FTPI upon admission presented IgG concentrations between 400 and 800 mg/dL.

The binomial logistic regression analysis included all continuous variables that showed statistical significance in the prior statistical tests, specifically rectal temperature, blood glucose concentration, and blood creatinine. Only blood glucose concentration reached statistical significance (*p* = 0.040), reinforcing the prognostic value of this parameter.

### 3.4. Comorbidities

Forty-four foals (72.1%) developed at least one type of comorbidity during the hospitalization. The development of comorbidities during the hospitalization was statistically associated with survival (*p* = 0.001) (Table 12) contributing to 40.1 greater odds for non-surviving.

Pneumonia affected 17/61 foals, of which 12/17 died. There was a significant association between this comorbidity and survival (*p* = 0.002), with pneumonia representing 5.14 times increased risk of death in this sample.

Nine foals developed septicemia. All the foals diagnosed with septicemia did not survive. Septicemia was statistically associated with survival (*p* = 0.01), with foals that develop this comorbidity during hospitalization being 11.8 times at greater risk of non-survival.

A total of 17/61 foals developed diarrhea, 6/61 foals developed colic, and 13/61 foals had an umbilical infection during hospitalization, however, no association with survival was detected.

The distribution of the different comorbidities between survivors and non-survivors is represented in Table 13.

## 4. Discussion

Even though NE is commonly reported, information regarding prognostic indicators in the scientific literature is scarce. The present work provides an insight into the value that some simple and accessible clinical and laboratory parameters can help predict the outcome of foals with NE upon admission. It also evaluates the impact that comorbidities developing during hospitalization may have on these foals.

The overall survival rate of the foals with NE and associated comorbidities presented in our sample (57.4%) is lower than the 70 to 80% reported in recent literature regarding foals with NE [3,16,24,25]. However, some authors mention that these high survival percentages refer to foals with NE but without the presence of complications [1], and in fact, in our sample, all the foals that did not develop comorbidities survived. Our study included 72.1% of foals that developed comorbidities which likely contributed to the lower survival rate. Furthermore, this study relied on medical records within a large time frame (2000 to 2022), presenting a tendency for older cases to have higher mortality than more recent ones. Several factors may be related to this event, such as an increase in knowledge about equine neonatology over the last few decades and an increase in the experience of clinical staff in this field. Nevertheless, most of the foals that did not develop any kind of comorbidity during hospitalization survived.

Regarding the medical history of the foals and mares, none of the evaluated factors showed an association with the outcome, which agrees with some studies and disagrees with others. The fact that we had a small number of premature foals and mares with placental problems and diseases during gestation may have contributed to this since Gold et al. 2016 [10] found an association between these variables and non-survival. Regarding the sex of the foals, Lyle-Dugas et al. 2017 [9] did not find any association with survival rate either. Prematurity is a condition that can predispose foals to acquire some clinicopathological signs that may be similar to NE, such as impairment of temperature and glucose regulation as well as renal, gastrointestinal, and respiratory dysfunction. However, some of the etiological factors related to prematurity are the same that cause NE, namely diseases during pregnancy and placental abnormalities. This may explain why some premature foals have concomitantly NE. Furthermore, some authors consider prematurity as a risk factor for the development of NE since it leads to a state of decreased tissue oxygenation and blood flow [3,12,21]. Regardless, the inclusion of premature foals with NE in this study may have created a bias in the interpretation of clinical and laboratory parameters, as these alterations could be related to the condition of prematurity rather than NE, being a limitation of the study.

Regarding clinical parameters, rectal temperature upon admission seems to have an important role in the outcome of these foals, since hypothermic foals presented greater odds of non-surviving. This was an expected finding since the presence of hypothermia in neonatal foals has been previously associated with non-survival in several studies [26,27]. Nevertheless, these studies included critically ill foals that presented with a wide range of diseases upon admission being non-specific for neonatal encephalopathy. Giguère et al., 2017 [27] also reported significant differences in the means of rectal temperature between survivors (higher mean) and non-survivors (lower mean), in accordance with our results. The same author also demonstrated that hypothermic neonatal foals had 6.24 greater odds of dying than the normothermic ones, which is higher than the 4.85 odds of non-surviving found in our sample. A possible justification is that Giguère et al., 2017 [27] evaluated neonatal foals that presented a wide range of diseases upon admission, being unspecific for NE, and evaluated clinical and laboratory parameters during hospitalization, which together may have influenced the results since these foals presented a more compromised clinical stage. In contrast, the clinicopathological features assessed in our study were recorded solely at the time of admission, when these foals had not yet exhibited significant changes beyond NE. Any additional comorbidities emerged afterward, during their hospitalization. Body temperature was also associated with survival in Dembek et al., 2014 [28] with the survivors group presenting a significantly higher temperature mean compared with non-survivors group, once more corroborating our results.

Upon admission, most foals (83.3%) presented recumbency. This clinical feature can have a role in the development of some comorbidities as stated by Perina et al., 2024 that reported a statistically significant association between recumbency and the development of umbilical diseases [29]. Our findings are similar to the results reported by Lyle-Dugas et al., 2017 [9] which found that recumbency was associated with non-survival and that foals with recumbency had 9.18 greater odds of dying, being higher than the likelihood reported in the present work (6.84 greater odds to die).

Regarding seizures, our results are in line with those of other studies such as Gold et al., 2016 [8], which found a significant association between the presence of seizures and the death of foals with NE. However, this study reported that foals with NE and seizures were 24 times more likely to die, which is much higher than the probability we found (4.14 times more likely to die). In humans, seizures are also associated with a worse prognosis [30]. Seizures increase oxygen and glucose consumption which can culminate in a more severe or even irreversible cerebral injury [1].

Blood glucose is a common parameter evaluated in several studies regarding newborn foals [9,22,27,28]. However, most of these studies are performed in critically ill foals, with various concomitant diseases. Hypoglycemia is common in neonatal foals given their low glycogen stores, particularly if they suffered significant stress at birth or if they have difficulties nursing. If prolonged, severe hypoglycemia can be life-threatening [12]. Hollis et al., 2008 [22] reported that, upon admission, 29.1% of the critically ill foals had blood glucose concentrations within the normal range, 36.5% had hyperglycemia, and 34.4% had hypoglycemia. Although our work presented a similar percentage of foals within the normal range (32.7%), we had more foals with hypoglycemia (51%) and just a few foals with hyperglycemia (16.3%). Nevertheless, Hollis et al., 2008 [22] reported a significant association between hypoglycemia at admission and death, in accordance with our findings. Foals that did not survive also had lower mean blood glucose concentrations at admission, which once more is in accordance with our results. Dembek et al., 2014 [28] also reported a significant difference in the blood glucose medians between survivors and non-survivors, in a sample of critically ill foals, somewhat corroborating our results. A feature reported by Hollis et al., 2008 [22], but not found in our study, was an association between hypoglycemia and septicemia. Perhaps the fact that Hollis included foals with different disorders may have contributed to this difference. Although the prevalence of hyperglycemia and its association with outcomes in foals is not well documented, there is a general acceptance that it is common and that it could be associated with poor outcomes [31]. Once more, Hollis et al., 2008 [22] did not find any association between low or moderate hyperglycemia and survival. However, extreme hyperglycemia was associated with a poorer prognosis. In our sample, the low number of foals presenting hyperglycemia did not allow us to perform any kind of interpretation. In our work, besides association tests, a multivariate test was carried out to assess the influence that multiple variables in conjunction could have on the survival of these foals. Blood glucose concentration was the only variable that stood out and reached statistical significance, underlining the importance of measuring this parameter during admission, giving it potential as a prognostic factor.

Transient high creatinine concentrations are frequently found in newborn foals that underwent hypoxic episodes during parturition, or in cases where maternal or placental abnormal conditions are reported [32]. Creatinine concentrations tend to decrease more than 50% in 24 h, however, this may not happen in some cases. An increase in creatinine concentrations on these foals may also be a consequence of hypovolemia with subsequently decreased renal perfusion or a result of nephrotoxic drugs, which, if not solved promptly, may lead to death [32]. A previously retrospective study that included 78 foals with NE reported that hypercreatinemia was one of the most common (32%) laboratory abnormalities found [7]. Even so, this percentage is lower than in our sample (61.8%). Although the same abnormality was common in Lyle-Dugas et al., 2017 [9]’s work, no association with survival was found. Similarly, Chaney et al., 2010 [32] did not find any significant difference in the creatinine means between survival and non-survival foals admitted at the hospital. As such, both articles presented contrasting results when compared with our work. Nevertheless, Dembek et al., 2014 [28] found significant differences in the creatinine median between survival and non-survival foals (2.3 and 4.3 mg/dL, respectively) being in line with our results, although we found slightly lower medians (1.95 and 3 mg/dL respectively).

According to McKenzie 2018 [12], anemia is a common finding in premature foals as well as hypoglycemia. Although in this study there was no association between these mentioned variables, anemic foals presented greater odds of dying. Similarly, Giguère et al., 2017 [27] reported that low red blood cell count was associated with non-survival.

Regarding FPTI, there is still some controversy, with some studies finding that foals presenting FPTI were less likely to survive [14], while others did not find any association [26]. In our study, no association was found either. One possible reason for this discrepancy between studies may be related to the IgG levels of the foals included in these studies. In our study, all the foals had IgG levels between 400 mg/dL and 800 mg/dL, which is considered a partial failure of the transfer of immunity and not a total failure. This condition, unlike total failure, is easily reversible in most cases and may not have any major consequences for the foal [12], which may have contributed to the lack of statistical association between the presence of this condition and death.

The other clinical and laboratory parameters evaluated during admission, like heart rate, respiratory rate, total protein, and BUN, did not show an association with survival. This is consistent with some previous reports [9,26,27,33].

Most of the foals included in this study developed at least one comorbidity during hospitalization. This finding is in line with the literature which mentions that, given the debilitated state of many of these foals, worsening of their clinical condition is common [1].

Pneumonia is a well-known cause of morbidity and mortality in foals since it increases the risk of sepsis. Depending on the severity, the respiratory distress also makes it difficult for the foal to feed and contributes to hypoxia [12]. Therefore, the association found in our study between the development of this comorbidity and mortality was expected and it is in line with several previous studies [9,12,27,34]

Regarding septicemia, the present study stands out from others by presenting a few cases of foals that developed septicemia (13.4%). Despite septicemia being one of the most common comorbidities in foals and undoubtedly the leading cause of mortality, the fact that the foals included in this study were admitted early and did not have a complete failure of passive transfer of immunity (FPTI) (<400 mg/dL), may have been factors that significantly impacted the low prevalence of this comorbidity. These foals had the opportunity to receive immediate therapy at all levels, with a rapid normalization of IgG levels and, consequently, of their immunity. Furthermore, the fact that the presence of sepsis on admission was considered an exclusion criterion in this study may have contributed to the low number of foals with this comorbidity. Nevertheless, all the foals that developed sepsis died, underlying the results of other studies, which also found an association between septicemia and non-survival [9,10,27].

The retrospective nature of the study led to some limitations. Not all the medical records were completed and the time frame between clinical cases could have some impact on the outcome of the foals given the differences in the diagnostic and therapeutical skills.

## 5. Conclusions

This study provides some evidence that certain basic clinical and laboratory parameters that are easily accessible to hospital and field clinicians can be used as predictors of outcomes in foals with neonatal encephalopathy. Special emphasis is placed on the presence of hypothermia, hypoglycemia, high creatinine levels, and anemia as prognostic factors for these foals.

## Figures and Tables

**Table 1:** Rectal temperature upon admission in survivors and non-survivors (*n* = 49).

	Survival			
Rectal Temperature	Survivors	Non-Survivors	Total	*p*
Normothermic	14	4	18	
Hypothermic	13	18	31	**0.015**
Total	27	22	49	

**Table 2:** Heart rates upon admission in survivors and non-survivors (*n* = 47).

	Survival			
Heart Rate	Survivors	Non-Survivors	Total	*p*
Normal	12	16	28	
Bradycardia	6	2	8	**0.52**
Tachycardia	8	3	11	
Total	26	21	47	

**Table 3:** Respiratory rates upon admission in survivors and non-survivors (*n* = 43).

	Survival			
Respiratory Rate	Survivors	Non-Survivors	Total	*p*
Normal	7	5	12	
Bradypnea	15	8	23	**0.115**
Tachypnea	2	6	8	
Total	24	19	43	

**Table 4:** Anemia in survivors and non-survivors (*n* = 61).

	Survival			
Anemia	Survivors	Non-Survivors	Total	*p*
No	33	20	53	
Yes	2	6	8	**0.04**
Total	35	26	61	

**Table 5:** Total protein concentration in survivors and non-survivors (*n* = 47).

	Survival			
Total Protein	Survivors	Non-Survivors	Total	*p*
Normal	19	19	38	
Hypoproteinemia	1	2	3	**0.613**
Hyperproteinemia	4	2	6	
Total	24	23	47	

**Table 6:** Presence of recumbency at admission in survivors and non-survivors (*n* = 61).

	Survival			
Recumbency	Survivors	Non-Survivors	Total	*p*
No	8	0	8	
Yes	27	26	53	**0.009**
Total	35	26	61	

**Table 7:** Presence of seizures in survivors and non-survivors (*n* = 61).

	Survival			
Seizures	Survivors	Non-Survivors	Total	*p*
No	29	14	43	
Yes	6	12	18	0.022
Total	35	26	61	

**Table 8:** Blood glucose concentrations in survivors and non-survivors (*n* = 49).

	Survival			
Blood glucose	Survivors	Non-Survivors	Total	*p*
Normoglycemic	14	2	16	
Hypoglycemic	8	17	25	**0.002**
Hyperglycemic	3	5	8	
Total	25	24	49	

**Table 9:** Blood creatinine concentration in survivors and non-survivors (*n* = 34).

	Survival			
Creatinine	Survivors	Non-Survivors	Total	*p*
Normal	10	3	13	
High	7	14	21	**0.013**
Total	17	17	34	

**Table 10:** BUN values at admission in survivors and non-survivors (*n* = 40).

	Survival			
BUN	Survivors	Non-Survivors	Total	*p*
Normal	7	8	15	
High	12	13	25	**0.658**
Total	19	21	40	

**Table 11:** FTPI at admission in survivors and non-survivors (*n* = 61).

	Survival			
FTPI	Survivors	Non-Survivors	Total	*p*
No	11	9	20	
Yes	24	17	41	**0.793**
Total	35	26	61	

**Table 12:** Development of comorbidities in survivors and non-survivors (*n* = 61).

	Survival			
Comorbidities	Survivors	Non-Survivors	Total	*p*
No	16	1	17	
Yes	19	25	44	**0.001**
Total	35	26	61	

**Table 13:** Association between different comorbidities developed during hospitalization of foals with NE and survival.

		Survival		*p*
Comorbidities		Survivors	Non-Survivors	
**Pneumonia**	**No**	30	14	0.002
	**Yes**	5	12	
**Septicemia**	**No**	32	20	0.01
	**Yes**	3	6	
**Diarrhea**	**No**	25	19	1
	**Yes**	10	7	
**Colic**	**No**	31	24	1
	**Yes**	3	3	
**Umbilical infection**	**No**	29	19	0.528
	**Yes**	6	7	

## Data Availability

Data are contained within the article and Supplementary Materials.

## Supplementary Materials

The following supporting information can be downloaded at: https://www.mdpi.com/article/10.3390/vetsci11110534/s1, Table S1: Epidemiological and clinical data of foals and mares.
