# Supplementary material for: Prognostic Value of Clinicopathological Parameters Assessed During Admission of Foals with Neonatal Encephalopathy and Comorbidities Developed During Hospitalization
Source: Vet Sci. 2024 Nov 1;11(11):534. doi: 10.3390/vetsci11110534 (PMC11598894; doi:10.3390/vetsci11110534)
Supplement: Supplementary file 1 [file vetsci-11-00534-s001.zip › vetsci-3203376-supplementary.pdf]

Table S1: Epidemiological and clinical data of foals and mares

Variable		Survivors	Non-survivors	*p*
**Foals gender**	*Male*	15	14	0.611
	Female	19	13	
**Foals age at admission (in hours)**	*Median (IQR)*	20 (30.2)	18 (25.8)	0.852
**Time since presentation of clinical signs**	*Median (IQR)*	13 (25.3)	15 (18.0)	0.261
**Prematurity**	*Yes*	5	8	0.209
	No	30	18	
**Mares age**	*Median (IQR)*	12 (7.5)	12 (10.5)	0.937
**Placental diseases**	*Yes*	2	1	0.715
	No	33	25	
**Dystocia**	*Yes*	10	7	1.00
	No	26	18	
**Diseases during pregnancy**	*Yes*	2	3	0.412
	No	33	23	
